# Cross-Modal Supervised Human Body Pose Recognition Techniques for Through-Wall Radar

**DOI:** 10.3390/s24072207

**Published:** 2024-03-29

**Authors:** Dongpo Xu, Yunqing Liu, Qian Wang, Liang Wang, Qiuping Shen

**Affiliations:** 1 School of Electronics and Information Engineering, Changchun University of Science and Technology, Changchun 130022, China; 2019200085@mails.cust.edu.cn (D.X.); 2019100528@mails.cust.edu.cn (Q.W.); sqp@mails.cust.edu.cn (Q.S.); 2 Jilin Provincial Science and Technology Innovation Center of Intelligent Perception and Information Processing, Changchun 130022, China; 3 Intelligent Perception and Processing Technology Laboratory, Beijing 100124, China; wangliang002@cust.edu.cn

**Keywords:** through-wall radar, target pose recognition, deep learning, cross-modal supervision, machine learning

## Abstract

Through-wall radar human body pose recognition technology has broad applications in both military and civilian sectors. Identifying the current pose of targets behind walls and predicting subsequent pose changes are significant challenges. Conventional methods typically utilize radar information along with machine learning algorithms such as SVM and random forests to aid in recognition. However, these approaches have limitations, particularly in complex scenarios. In response to this challenge, this paper proposes a cross-modal supervised through-wall radar human body pose recognition method. By integrating information from both cameras and radar, a cross-modal dataset was constructed, and a corresponding deep learning network architecture was designed. During training, the network effectively learned the pose features of targets obscured by walls, enabling accurate pose recognition (e.g., standing, crouching) in scenarios with unknown wall obstructions. The experimental results demonstrated the superiority of the proposed method over traditional approaches, offering an effective and innovative solution for practical through-wall radar applications. The contribution of this study lies in the integration of deep learning with cross-modal supervision, providing new perspectives for enhancing the robustness and accuracy of target pose recognition.

## 1. Introduction

Traditional through-wall radar methods for target pose recognition primarily rely on directly extracting target pose information from radar echo signals. However, this approach is plagued by issues such as information loss, difficulty in capturing pose diversity, complex feature engineering, and inadequacy in adapting to complex scenarios. In the field of computer vision, the estimation of human body pose is commonly categorized into two approaches: top-down and bottom-up. The top-down approach involves initially detecting each person in the image and then applying a single-person pose estimator to extract keypoint information, as evidenced by prior works [1,2,3,4]. In contrast, the bottom-up approach, as demonstrated in previous studies [5,6,7], first detects all keypoints in the image and then associates keypoints of the same target through a post-processing procedure. Radar echo images often struggle with clear target identification but can provide limited information about the positions of target keypoints. Furthermore, compared to traditional RGB-D sensors and external sensors like Vicon [8,9], radar signals have the advantage of being less susceptible to interference from walls and other opaque structures.

In this study, a bottom-up approach is adopted, building upon existing methods, with a focus on cross-modal and multimodal modeling to explore complementary information matching or cross-modal information transfer. By integrating camera and radar information and leveraging deep learning frameworks, this paper achieves more accurate and robust through-wall radar target pose recognition. The objective of this innovative approach is to overcome the limitations of traditional methods, comprehensively utilizing multimodal information to enhance target pose recognition performance in complex environments.

Recent academic research in the field of human pose recognition has predominantly concentrated on overall body position localization, monitoring walking speed, tracking chest movements to extract respiratory and heartbeat information, and tracking arm movements for specific gesture recognition. Systems such as the RF-Capture designed by Adib et al. [10] offer a rough description of a person’s position behind a wall based on signals detected at multiple time points from different body parts. However, previous research showed limitations in accurately estimating body poses or precisely locating various key body parts, thereby falling short of achieving comprehensive and accurate human pose estimation.

This study aimed to bridge this research gap by achieving comprehensive and precise estimation of human body pose through the integrated consideration of information from multiple body parts. To effectively separate reflection signals from different objects, advanced waveform modulation techniques such as FMCW (frequency-modulated continuous wave) and SFCW (step frequency continuous wave) are commonly employed. FMCW technology modulates the frequency of the transmitted signal, enabling the calculation of the target’s distance by measuring the frequency difference in the returned signal. In FMCW radar, the frequency of the continuous wave signal changes linearly or nonlinearly, typically increasing over time. When this signal is mixed with the signal reflected from the target, the distance to the target can be determined by measuring the frequency difference. SFCW radar, on the other hand, transmits a series of continuous wave signals with discrete frequency steps and then measures the phase and amplitude of the returned signal to obtain target information.

In recent years, alongside research focusing on Doppler-based human motion detection in indoor environments [11], there have also been studies on describing human representation information through SAR imaging [12]. However, these studies primarily focused on human detection. The core method proposed in this paper still lies in the three-dimensional imaging of human targets, followed by gesture recognition, as three-dimensional results offer richer and more intuitive information.

**Figure 1:**
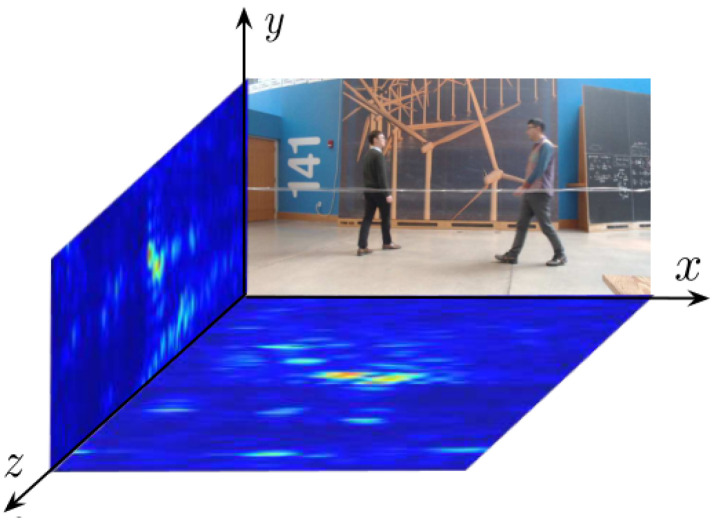
Radar heatmaps and camera RGB images.

This study drew inspiration from the approach presented in the relevant literature [13,14] to construct a radar system. The system generates SFCW signals and is equipped with a two-dimensional antenna array. The input data of the system are presented in the form of a two-dimensional heatmap, comprising horizontal and vertical heatmaps. The horizontal heatmap represents the projection of signals on a plane parallel to the ground, while the vertical heatmap represents the projection of signals on a plane perpendicular to the ground, with red indicating higher values and blue indicating lower values, as illustrated in Figure 1. Since radar signals are in complex in form, each pixel in the heatmap contains both real and imaginary parts. Thirty pairs of such heatmaps can be generated per second, providing a rich data foundation for subsequent pose estimation.

## 2. Preprocessing of Target Signals behind Walls

Through-wall radar for human body pose recognition represents a critical application within radar systems, offering extensive integration opportunities with computer vision technology in areas such as surveillance, behavior recognition, and disaster rescue. The process of human body pose recognition primarily focuses on identifying crucial joint locations (e.g., arms, legs) and key points (e.g., head, torso). Unlike cameras, radar encounters challenges with occlusion, particularly when obstructed by walls. Many existing studies addressing occlusion relied on deep learning for target generation. However, due to the inherent uncertainty associated with human body poses, this approach is susceptible to errors. With advancements in sensor technologies, the fusion of data from multiple sensors has emerged as a promising direction to mitigate occlusion-related challenges in pose recognition. The method studied for human body pose estimation using through-wall radar, as explored in this paper, holds relevance for potential applications in other visual recognition scenarios [15,16,17].

### 2.1. Radar Antenna Parameter Design

This section details the design of the antenna unit, rooted in radar array design theory and adopting the virtual aperture planar array format to achieve the UWB radar signal transmitting and receiving functions. In this study, adjustments were made to the number and geometry of the transmitting and receiving antennas to evaluate the impact of various array configurations on the 3D imaging performance. This process aimed to offer robust insights into determining the ultimate configuration of the virtual aperture planar array.

In examining UWB antenna forms, various common types were explored, encompassing monopole antennas, dual-cone dipole antennas, wide-slot antennas, logarithmic spiral antennas, and logarithmic periodic antennas, with an analysis of their respective characteristics. These antennas are adept at emitting broadband RF signals and exhibit distinct attributes. Notably, in the realm of through-wall radar system equipment development, the aspect of weight is often underestimated. When selecting antenna materials, it becomes imperative to consider the physical properties of the cables, including their volume, cross-sectional area, and weight. With these considerations in mind, this study opted for microstrip patch antennas as the foundational material. Furthermore, in designing antenna units, it becomes paramount to address both wideband performance and electrical properties, while ensuring applicability across various scenarios. To this end, the proposed solution involves employing microstrip butterfly-shaped antennas with a back cavity design.

Antenna Performance Parameters:

Operating frequency range: 1.9 GHz–2.9 GHz; antenna type: microstrip butterfly-shaped resonator antenna; VSWR (voltage standing wave ratio): <2; Gain: ≥5 dBi; front-to-back ratio: ≥10 dB; beamwidth: $\mathrm{Azimuth}\geq 60^{\circ }$, $\mathrm{Elevation}\geq 45^{\circ }$; unit size: ≤7.5 × 7.5 × 6.5 cm; array scale: T10R10.

**Figure 2:**
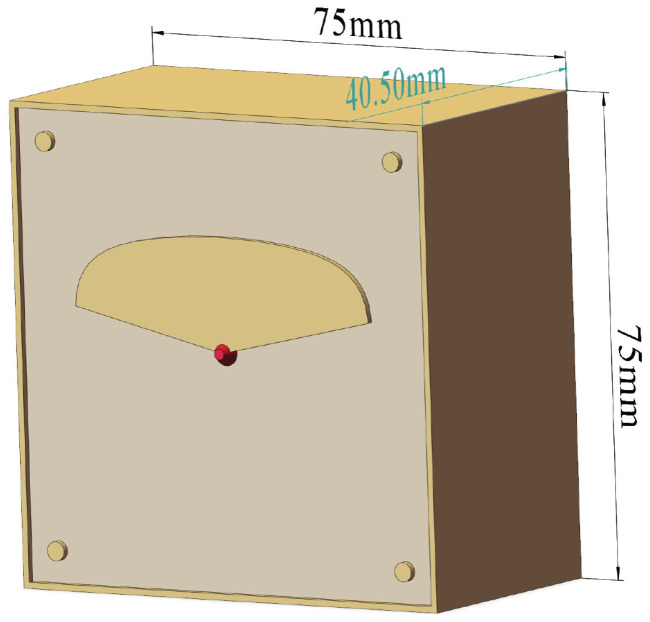
Antenna simulation model.

The simulation model for the wideband butterfly-shaped resonator antenna is illustrated in Figure 2.

**Figure 3:**
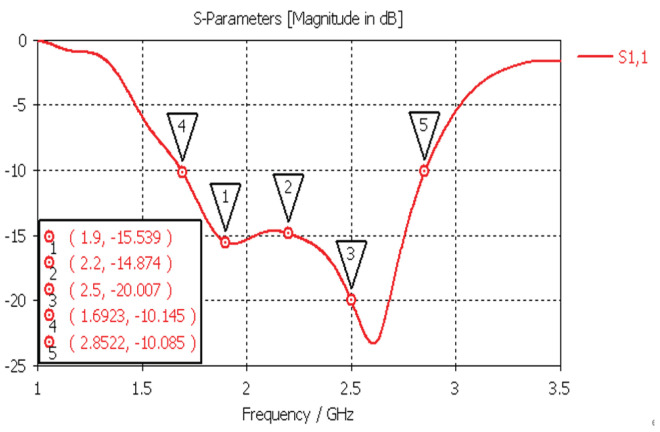
Simulation results for antenna return loss (S11).

**Figure 4:**
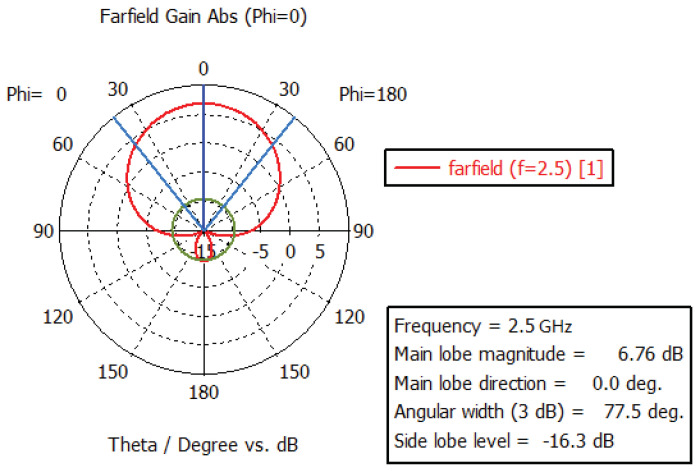
Simulation results at typical frequency points for the antenna.

From the Figure 3, it can be observed that the return loss (S11) of the antenna remained below −10 dB (equivalent to VSWR < 2) across the frequency range of 1.7 GHz to 2.85 GHz. As indicated in Figure 4 below, the designed antenna exhibited characteristics such as gain and 3 dB beamwidth.

The radar system adopted a stepped-frequency continuous-wave signal regime, representing a novel radar approach based on through-wall radar three-dimensional imaging technology. Compared to traditional pulse radar systems, the stepped-frequency continuous-wave signal regime offers a higher signal-to-noise ratio and lower power consumption. Additionally, this paper leveraged ultra-wideband (UWB) technology, where the antenna unit boasts a wide bandwidth, enabling higher resolution and finer imaging effects. To meet the integration demands of the radar system, a planar structure was utilized in the antenna design. Compared to traditional three-dimensional antennas, planar antennas offer lower manufacturing costs, a simpler structure, and a smaller size, thus enhancing the radar system’s integration. Moreover, the radiation characteristics of planar antennas and the design of antenna arrays meet the system’s requirements for high resolution and low side lobes. In summary, the design of the stepped-frequency continuous-wave signal regime and UWB antenna based on through-wall radar three-dimensional imaging technology achieved higher imaging accuracy and a smaller system size, showcasing superior practical performance and promising broad application prospects.

### 2.2. Radar Array Structure Configuration

#### 2.2.1. Antenna Array Configuration Platform

This section explores the design of the antenna array configuration platform, aiming to utilize a two-dimensional sparse planar array based on multi-transmitter multi-receiver technology to achieve three-dimensional imaging. Various factors such as imaging quality and system complexity were taken into account during the design process. The primary focus regarding imaging quality lay in achieving optimal focusing quality and positioning accuracy. System complexity was comprehensively considered by imposing constraints on the number of elements, aperture size, and minimum element spacing, while employing traditional sparse array design methods.

**Figure 5:**
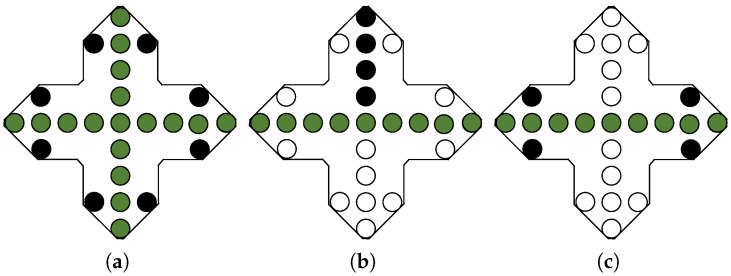
Virtual aperture planar array structure. (**a**) crowded face mask (math.). (**b**) Multi-baseline sparse surface array. (**c**) Single-baseline sparse surface array.

To establish a mathematical relationship model between aperture parameters and image parameters, optimization procedures involving aperture shape, size, element spacing, number of elements, and aperture formation time were carried out. This optimization design endeavored to attain high-quality imaging, while maintaining reasonable control over system complexity to meet the practical application requirements. To validate the effectiveness of the antenna array topology, a virtual aperture sparse array was formed by extracting certain transmit-receive antenna units based on the virtual dense aperture array. During the evaluation of the different extraction schemes for obtaining multi-baseline or single-baseline three-dimensional imaging performance, consideration was also given to the system weight. Figure 5 illustrates an approach for obtaining validation data based on a virtual dense aperture array, where the black circles, green circles, and white circles represent the transmit element, receive element, and idle element, respectively.

#### 2.2.2. Switch Matrix Module Design

The switch matrix encompasses two integral components: the transmission switch matrix and the reception switch matrix, each characterized by specific metrics:

Transmission switch matrix metrics and Reception switch matrix metrics:

(1) Operating frequency range: 1.9 GHz–2.9 GHz;
(2) channel selection paths: 1 out of 10;
(3) channel isolation: ≥45 dB.

The antenna array comprised 10 transmit and 10 receive channels, with operational procedures involving sequential switching from channel 1 to channel 10 for both transmission and reception. Upon completion of all 10 channel switches, the transmit channel transitions to channel 2, while the receive channels sequentially shift from channel 1 to channel 10. This iterative process results in a total of 100 channel switches.

In the design of the switch matrix, two considerations were paramount: the switch switching speed and switch isolation. Swift transitions of the switch are crucial to ensure brief durations at each frequency point, thereby facilitating a high scanning refresh rate. The designated switch switching speed was confined within a stringent timeframe of 100 ns. Simultaneously, switch isolation safeguards against unwanted inter-channel crosstalk during the reception of echoes, ensuring the fidelity of the resultant image.

**Figure 6:**
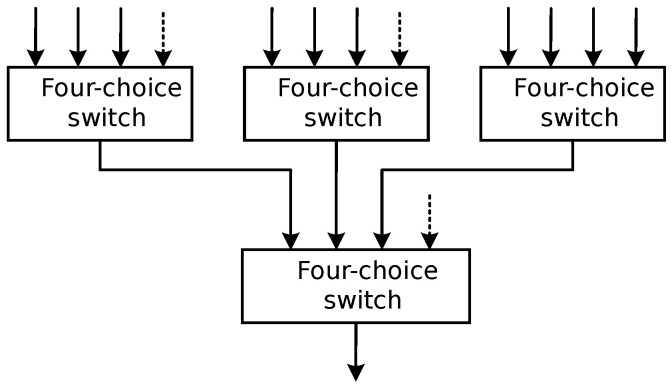
Switch matrix topology diagram.

Given the tenfold nature of both transmit and receive channels, and recognizing the inadequacy of a singular switch chip for simultaneously fulfilling all channel switching requisites while maintaining requisite isolation, the design adopted a judicious two-stage cascaded switch architecture, as depicted in Figure 6. Specifically, the transmit switch and receive switch designs remained aligned. However, before ingress into each receive switch, an essential low-noise amplifier (LNA) stage is incorporated. This augmentation ensures that the switch’s introduction exerts minimal impact on the overall system noise level. This strategic design approach harmonized switch array performance and isolation efficacy, ultimately achieving superlative radar image quality, while optimizing system noise control. The multi-baseline surface array method of acquisition was selected, and the wall material was a 12 cm brick wall (the actual thickness was 11.5 cm).

### 2.3. Preprocessing and RF Front-End Design

#### 2.3.1. Data Acquisition and Preprocessing Section

The data acquisition and preprocessing module are primarily responsible for collecting and preprocessing radar echo signals, encompassing tasks such as digital down-conversion, digital filtering, inter-channel calibration, pulse compression, and more. Once the data undergo preprocessing, they are uploaded to both the upper computer and the image processing module for further analysis.

Technical specifications of the data acquisition and preprocessing module:

(1) Sampling rate: ≥20 MHz;
(2) intermediate frequency: 15 MHz;
(3) A/D quantization bits: 16 bits.

Control commands and operational parameters of the system are transmitted from the signal processing unit to the RF front-end preprocessing board. For the downstream data flow: Initially, radar control commands and operational parameters are transmitted via Ethernet using the TCP protocol from the display control software, running on the display control computer, to the information processing unit. Upon receiving these commands and parameters from the display control, the information processing unit verifies and adjusts them as necessary before relaying corresponding instructions to the RF front-end preprocessing unit via USB. These instructions specify the working modes, frequency steps, and bandwidth steps. The RF front-end promptly confirms receipt of the command information.

For the upstream data flow: In sending data upstream, the RF front-end transfers echo data to the information processing unit via USB. Subsequently, the information processing unit forwards operational parameters and echo data to the display control and data storage unit through Ethernet.

The RF front-end module comprises three components: a frequency synthesizer, transmitter, and receiver. The technical specifications of the RF front-end section are as follows:

(1) Operating frequency range: covering 1.9 GHz–2.9 GHz;
(2) transmitter output power: +20 dBm;
(3) frequency step: 4 MHz/2 MHz;
(4) receiver intermediate frequency: 15 MHz.

#### 2.3.2. RF Front-End Module Section

**Figure 7:**
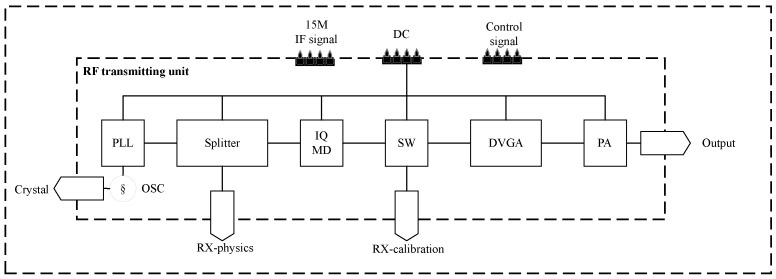
Block diagram of frequency synthesizer and transmitter design.

The frequency synthesizer and transmitter are pivotal components of this system. The frequency synthesizer’s primary role is to generate the SFCW local oscillator signal, while the transmitter is responsible for tasks such as quadrature upconversion, filtering, and amplification. To optimize the overall size and power consumption, this paper integrated the design of the frequency synthesizer and transmitter. The frequency synthesizer and transmitter comprise a crystal oscillator (serving as a common source for splitting), filters, a quadrature modulator, 1 × 2 RF switch (for toggling between calibration and transmission channels), digital attenuator, power amplifier, and more, as depicted in Figure 7.

**Figure 8:**
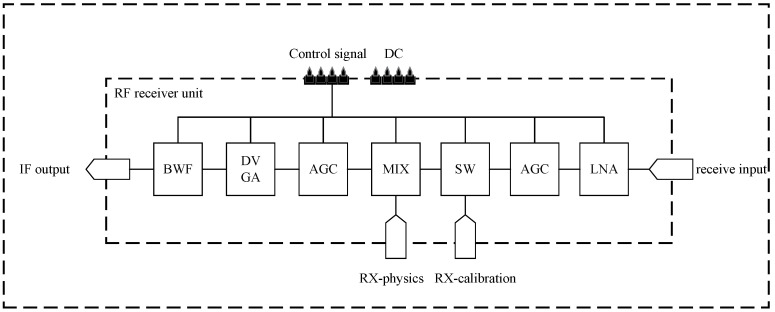
Receiver design schematic diagram.

The receiver is mainly composed of a low-noise amplifier, RF AGC, switch, mixer, IF AGC, digital gain amplifier, and bandpass filter, as shown in Figure 8 below.

**Figure 9:**
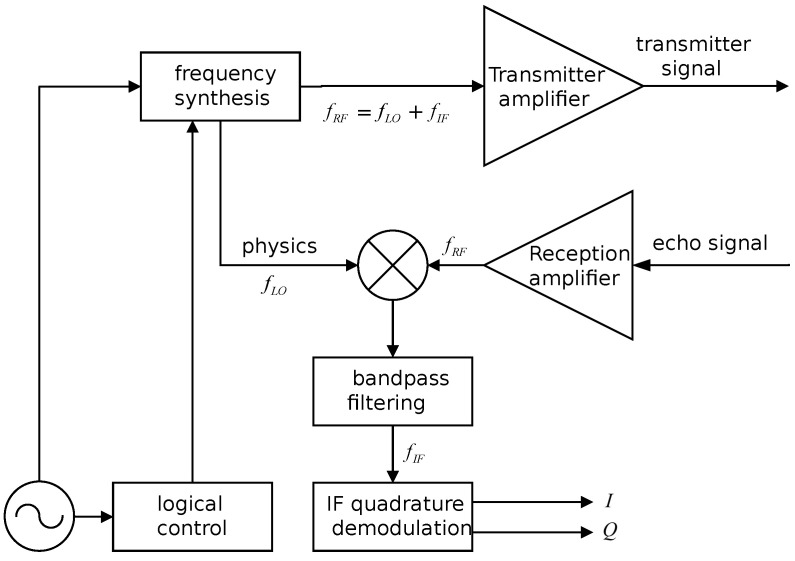
Schematic diagram of fixed-IF receiver.

The structure of through-wall radar receivers generally falls into two categories: zero-IF (intermediate frequency) receivers and fixed-IF receivers. Zero-IF receivers are capable of generating two signals: the in-phase signal and the quadrature signal. To tackle the challenge of image interference in zero-IF receivers, the receiver’s frequency can be set to zero. However, this introduces issues such as DC divergence and nonlinear effects in the digital domain, rendering target detection relatively challenging. Therefore, this paper adopted a fixed-IF receiver approach, as depicted in the schematic diagram in Figure 9. The fixed-IF receiver helps alleviate noise interference associated with zero-IF receivers.

The intermediate frequency (IF) quadrature demodulation is implemented in the digital domain, typically without the need for an external frequency synthesizer to provide the IF local oscillator signal. This approach is common in many radar receivers.

### 2.4. Imaging System Design

**Figure 10:**
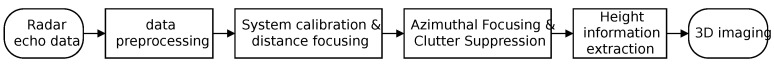
Three-dimensional through-wall imaging radar data processing flowchart.

The information processing unit is responsible for collecting, storing, and processing the radar echoes received by the receiver, including tasks such as three-dimensional imaging and clutter suppression. The data processing flow is illustrated in Figure 10.

(1) Two-dimensional imaging processing:

To accomplish range and azimuth focusing, this study primarily explored time-domain imaging algorithms (such as backprojection algorithms) and frequency-domain imaging algorithms (like range migration algorithms). Time-domain imaging algorithms can accommodate arbitrary array topologies but come with high computational complexity. On the other hand, frequency-domain imaging algorithms offer lower computational complexity but are usually confined to handling uniform arrays. Moreover, during data processing, attention was directed towards electromagnetic wave refraction and dispersion resulting from wall penetration, with the objective of enhancing the focusing quality and localization accuracy.

(2) Clutter suppression:

To achieve high signal-to-noise ratio radar images, methods for wall clutter suppression and ghost clutter suppression are studied. Wall clutter suppression methods include spatial filtering and singular value decomposition. Ghost clutter suppression methods involve frequency response enhancement and spatial position correlation.

During actual data processing, a flexible combination of the above imaging algorithms, height information extraction methods, and clutter suppression methods was determined based on real-world conditions. This ensured the final measured data imaging processing flow produced high-quality results. Furthermore, the effectiveness of each method could be verified through actual data imaging processing, laying the foundation for the subsequent research on three-dimensional through-wall imaging radar. The specific imaging system will be detailed in the following two sections.

(3) Height information extraction:

Height information is extracted to differentiate individuals with different postures. Multiple baseline tomography and single baseline interferometry methods are employed for height information extraction. Tomography methods use multiple virtual line arrays distributed in the height direction to extract target height information, while interferometry uses two virtual line arrays for angle interferometry, and subsequently obtains height information. Tomography has a stronger robustness but requires more antenna units and higher computational complexity. Interferometry is easier to implement for lightweight systems and may have larger errors when dealing with low signal-to-noise ratios and complex targets.

## 3. Modeling of Through-The-Wall Radar Imaging Systems

### 3.1. Imaging System Model

When radar signals propagate and encounter obstacles, the inhomogeneity of the medium causes the signals to reflect, refract, and scatter as the electromagnetic waves pass through the interface separating the two media. This results in the obtained sensor signal being less favorable for research and complicates the model simplification process. Therefore, when selecting an imaging algorithm, it is crucial to consider the refractive properties of electromagnetic waves between different media. Generally, the backprojection (BP) algorithm is chosen to process the signal and compensate for the signal refraction loss between different media. At the core of this algorithm lies the computation of delayed-superposition summation for each imaging point in the imaging region, which necessitates extensive data computation in the final 3D reconstruction process.

**Figure 11:**
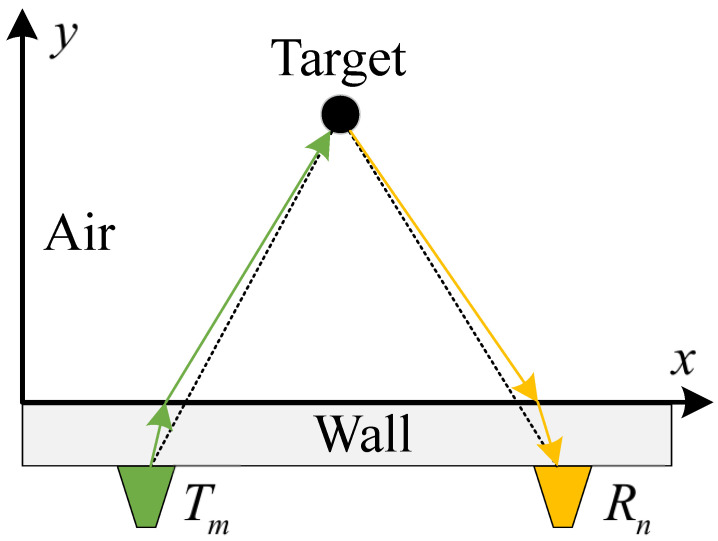
Schematic diagram of through-wall radar system.

As depicted in Figure 11, a wall-penetrating radar emits electromagnetic wave signals from a transmitter $T_{m}$ towards the wall. Upon encountering a target object, the signal reflects within a certain angle, forming an echo signal. This echo signal penetrates the wall again and is received by the receiving end, $R_{n}$, where it undergoes coherent superposition. A data space is defined in this space, and coherent focusing is applied to each data point within the data control, to accomplish 3D image reconstruction. The current challenges in through-wall radar imaging often revolve around three main points: (1) low resolution of imaging results due to limitations of the signal model; (2) the slow operational speed of the system stemming from the core principle of BP imaging; and (3) the interference of wall clutter, multipath clutter, and other noises, leading to poor robustness of the imaging process.

**Figure 12:**
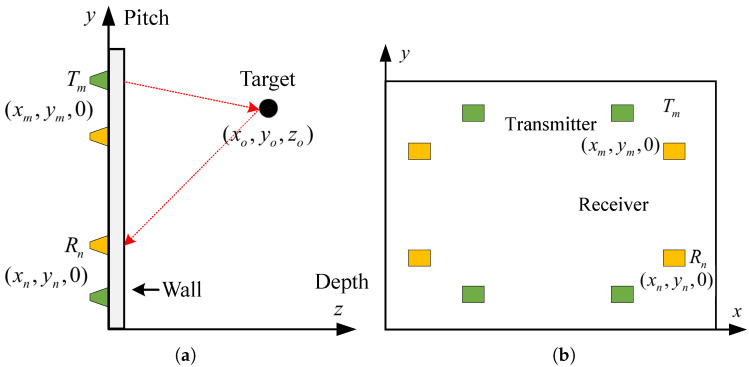
MIMO radar array imaging geometry. (**a**) left view. (**b**) front view.

The system in this paper utilizes multiple transceiver units arrayed using SFCW signals with continuously adjustable segmentation frequency. Switches are employed for time-division control of the antennas. Compared to SAR radar, the acquisition system presented herein boasts advantages such as a rapid acquisition time and unrestricted motion space, thereby rendering the system size and cost more manageable. An imaging diagram of the MIMO radar array is illustrated in Figure 12 below:

(a) is the left view and (b) is the main view. The entire imaging radar system contains M transmitting antennas and N receiving antennas. The *m*th transmitting antenna $T_{m}$ and the *n*th receiving antenna $R_{n}$ are located at $\left(x_{m},y_{m},0\right)$, $\left(x_{n},y_{n},0\right)$, where m=1,2,…,M, n=1,2,…,N. The form of the SFCW signal transmitted by the radar can be expressed as

(1) ST(t)=∑q=1Qe−j2π(f0+(q−1)Δf)trect(ttTT−q)

Δf is the step size of the SFCW radar, *Q* is the number of radar frequency points, and *T* denotes the time in each frequency band. At this time, the point $\left(x_{o},y_{o},z_{o}\right)$ is set in space, and for the *m*th transmitting antenna $T_{m}$, the *n*th receiving antenna $R_{n}$, and the *q*th frequency point $f_{q}=f_{0}+\left(q-1\right)\Delta f$, the echo signal of the target body can be written as

(2) $$S_{m,n,q}=\alpha \exp\left(-j2\pi f_{q}\tau_{mn}\right)$$

where $\tau_{mn}=\left(\sqrt{{\left(x_{o}-x_{m}\right)}^{2}+{\left(y_{o}-y_{m}\right)}^{2}+z_{o}^{2}}+\sqrt{{\left(x_{o}-x_{n}\right)}^{2}+{\left(y_{o}-y_{n}\right)}^{2}+z_{o}^{2}}\right)/c$ is the time delay of the received signal, *c* is the speed of light, and α is the target reflection coefficient. For a distributed target, the expression for the received signal can be expressed as the following equation:

(3) $$S_{m,n,q}=\underset{x_{o}y_{o}z_{o}}{\;\int \int \int \;}\alpha \left(x_{o},y_{o},z_{o}\right)e^{-j2\pi f_{q}\tau_{mn}}dx_{o}dy_{o}dz_{o}$$

For the SFCW-MIMO system within a sampling period, there are a total of M×N channels in the frequency domain echo data, which contain the amplitude and phase information and constitute the imaging data space. In this part of data control, at any point (x,y,z), the echo signal is given by Equation (3), and the signal pixel values in its space are given by Equation (4):

(4) $$I\left(x,y,z\right)=\sum_{q=1}^{Q}\sum_{m=1}^{M}\sum_{n=1}^{N}S_{m,n,q}e^{+j2\pi f_{q}\tau_{mn}^{*}},$$

where the time delay of the pixel points divided by the imaging area is $\tau_{mn}^{*}$:

(5) $$\tau_{mn}^{*}=\left(\sqrt{{\left(x-x_{m}\right)}^{2}+{\left(y-y_{m}\right)}^{2}+z^{2}}+\sqrt{{\left(x-x_{n}\right)}^{2}+{\left(y-y_{n}\right)}^{2}+z^{2}}\right)/c.$$

The relative scattering intensity value of the received signal can be obtained according to $\tau_{mn}^{*}$. For distributed targets, the coefficient of each pixel point in the reconstructed region can be expressed as Equation (6):

(6) $$I\left(x,y,z\right)=\underset{x_{0}y_{0}z_{0}}{\;\int \int \int \;}\alpha \left(x_{0},y_{0},z_{0}\right)\sum_{q=1}^{Q}\sum_{m=1}^{M}\sum_{n=1}^{N}e^{j2\pi f_{q}\left(\tau_{mn}^{*}-\tau_{mn}\right)}dx_{0}dy_{0}dz_{0}.$$

The equation above defines a pixel’s value in a data grid, enabling the creation of a 3D focus map by aggregating grid points. Closer alignment of the target with the pixel coordinates results in clearer imaging. To enhance a wall-penetrating radar’s range resolution and penetration, signal regimes often shift to SFCW and utilize BP imaging in the frequency domain for increased dynamic range and power.

The Nyquist theorem stipulates a sampling frequency exceeding twice the signal bandwidth, to prevent information loss in uniform sampling. However, for sparse signals, compressed sensing theory offers a novel sampling method using random matrices, achieving accurate signal reconstruction with minimal data. This theory finds wide application in signal sampling, image processing, radar imaging, and channel coding, relying on linear projection for precise reconstruction.

Traditional through-wall radar employs time-domain sampling, necessitating higher speeds and storage due to signal complexity. Thus, frequency domain compressed sensing has gained traction, compressing data by extracting sub-bands, significantly reducing sampling rates, storage needs, and costs compared to time sampling.

In through-wall radar, frequency domain compressed sensing reduces data acquisition and processing costs, while enhancing imaging resolution and target detection depth. Careful parameter selection mitigates information loss, as spectral aliasing effects may occur due to discrete sub-band extraction. Future advancements in technology will likely broaden the application of frequency domain compressed sensing in through-wall radar, further improving efficiency and accuracy.

**Figure 13:**
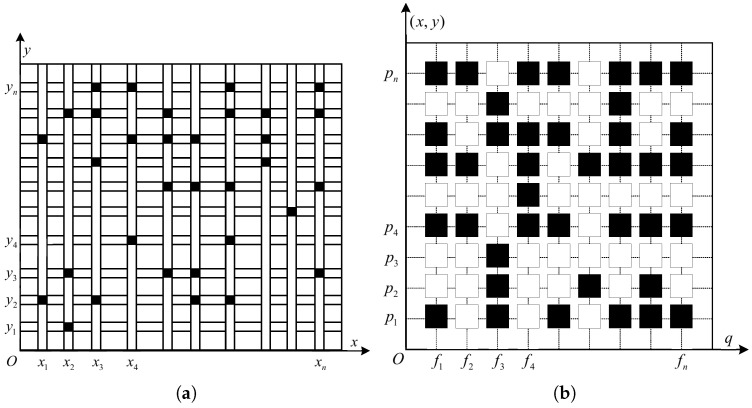
Schematic diagram of random measurement of frequency points. (**a**) Antenna measurement position. (**b**) Frequency measurement position.

In the practical engineering application of through-wall radar imaging, other RF signal interferences cannot be avoided in the space, and the sampling data of certain frequency points are difficult to obtain, i.e., the frequency signal of the target is also sparse in the space. Therefore, the selection of the measurement frequency points should include randomly selected geographic locations in the target area, which can enhance the diversity of the data and obtain more target information. Random measurements are used to select random frequency data at random aperture locations to enhance the robustness of the reconstruction results, and the case of joint compression in the spatial and frequency domains is used in the construction process of the measurement matrix Φ. The value of $\Phi_{p}$ changes with the value of *p*, i.e., measurements are performed at different aperture locations and different frequency points. The model is illustrated in Figure 13 below.

### 3.2. Through-Wall Radar Clutter Suppression Techniques

#### UWB Virtual Aperture Imaging Method

Clutter suppression techniques in through-wall radar systems play a pivotal role in enhancing imaging quality. Among these, synthetic aperture radar (SAR) stands out as a rapidly evolving radar technology capable of achieving high-resolution 2D imaging by transmitting broadband signals, while forming arrays in the azimuthal direction. However, SAR radar encounters several practical constraints, including challenges in achieving forward-looking imaging and accurately focusing on stationary and moving targets simultaneously. Although some researchers have proposed methods like dual-base station SAR radar for forward-looking imaging, extensive research is required to address the many potential issues.

In numerous application scenarios, there is an urgent need for perspective imaging of static and moving targets, or even simultaneous imaging. For instance, in self-driving vehicles, real-time assessment of obstacles ahead such as rocks or deep holes is crucial. Similarly, in military operations, visual detection of objects behind walls and the movement of personnel is essential. However, conventional SAR radars face limitations such as the large number of arrays, heavy weight, large size, and system complexity inherent in real aperture imaging. To tackle these challenges, virtual aperture imaging has emerged as a promising solution strategy. Virtual aperture imaging relies on multiple transmitters and receivers, constructing a virtual array between each pair of transceiver antennas to achieve target imaging. This method circumvents many limitations of traditional real aperture imaging and provides an effective means of meeting diverse imaging requirements.

In multi-transmitter-multi-receiver arrays with transceiver split mode, the conventional SAR imaging method becomes inapplicable, and the Backprojection (BP) algorithm is employed to achieve virtual aperture imaging. However, due to the limited number of actual ultra-wideband (UWB) antenna units, sparsity in virtual aperture imaging can lead to increased image sidelobes. To address this issue, the coherence factor method is employed for sidelobe suppression. The coherence factor method is a commonly used image processing technique aimed at suppressing sidelobes in an image, and it is computed using the following formula: coherence factor = actual imaging data/estimated imaging data.

(7) $$CF\left(x,y\right)=\frac{{\left|\;\int \int \;h\left(x,y\right)s_{1}\left(t,x_{A}\right)\sigma \left(t-2\sqrt{{\left(x-x_{R}\right)}^{2}+{\left(y-y_{R}\right)}^{2}}/c\right)dx_{A}dt\right|}^{2}}{N_{A}{\;\int \int \;\left|h\left(x,y\right)s_{1}\left(t,x_{A}\right)\sigma \left(t-2\sqrt{{\left(x-x_{R}\right)}^{2}+{\left(y-y_{R}\right)}^{2}}/c\right)\right|}^{2}dx_{A}dt}$$

where CF(x,y) is the value of the coherence factor at position (x,y). $N_{A}$ is the signal amplitude, denoting the signal strength. $dx_{A}$ denotes the signal space, and dt denotes the temporal resolution, which denote spatial minutiae and temporal minutiae, respectively. The coherence factor is calculated using the energy of the signal divided by the product of the spatial and temporal minutiae of resolution, describing the coherence of the signal over a specific spatial and temporal range.

This section explores coherence factor weighting as a means for processing imaging results effectively, aiming to suppress the generation of sidelobes. In virtual aperture imaging, the utilization of an ultra-wideband (UWB) virtual aperture surface array deviates from the traditional virtual aperture line array. To address this variation, this paper further explores the backprojection (BP) algorithm based on the UWB virtual aperture surface array and its sidelobe suppression method. Through experiments, the influence of sidelobes in virtual aperture linear array imaging was effectively mitigated by comparing actual imaging data with estimated imaging data after coherence factor weighting, consequently leading to a significant enhancement in imaging quality.

**Figure 14:**
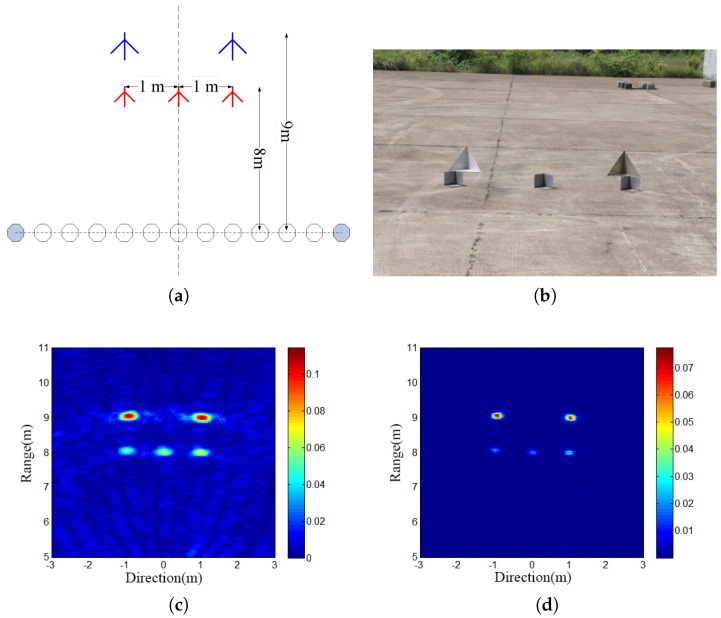
Virtual aperture line array imaging results. (**a**) Test scenarios. (**b**) Target photo. (**c**) Imaging results. (**d**) Clutter suppression results.

The coherence factor-weighted virtual aperture imaging method successfully mitigated the sparsity issue stemming from the limited number of actual UWB antenna units and efficiently suppressed sidelobes in the image. Implementing this method aided in enhancing the resolution and accuracy of virtual aperture imaging, thus furnishing a more dependable foundation for subsequent data analysis and target attitude recognition. The imaging results of the virtual aperture line array are depicted in Figure 14.

Comparison between actual imaging data and estimated imaging data weighted by the coherence factor was conducted to evaluate the efficacy of this method. This research represents a significant advancement in virtual aperture imaging techniques, particularly in scenarios with a restricted number of ultra-wideband (UWB) antenna units, aiming to enhance imaging quality and contribute to the advancement of clutter suppression techniques for through-the-wall radar systems.

## 4. Methodology for Through-Wall Radar Human Body Pose Estimation

**Figure 15:**
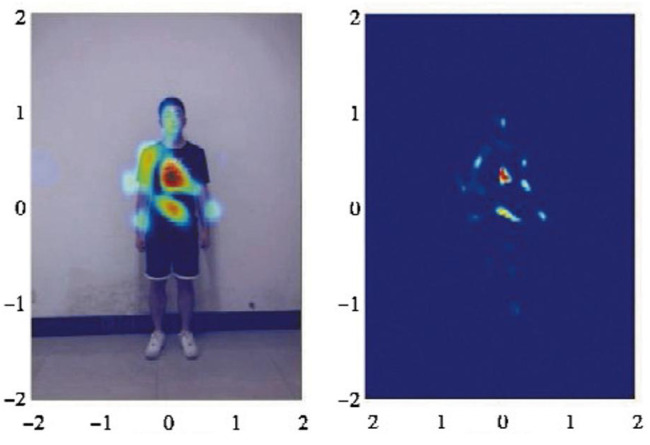
Radar heatmap.

Utilizing radar signals to delineate the pose of a human body target generates a heatmap, as depicted in Figure 15, which only vaguely outlines the human silhouette. For such datasets, viable approaches include semi-supervised learning and transfer learning. Semi-supervised learning entails training with a small labeled dataset augmented by a large unlabeled dataset. On the other hand, transfer learning leverages labeled data from an established domain, employing them in a new domain through transfer learning mechanisms, thereby mitigating the necessity for extensive labeling in the new domain.

This paper introduces a transfer learning-based methodology for through-wall radar human target pose recognition, designed to be adaptable across various visual recognition scenarios and serving as the foundational framework for an integrated radar system. This system enables posture analysis behind walls by decoding the spatial propagation characteristics of emitted radar radio frequency signals and extracting distinctive features of human subjects.

Utilizing cross-modal supervision, the model initially learns posture estimation tasks in the source domain of camera information and subsequently transfers this acquired knowledge to the target domain of radar information. By leveraging supervised learning on camera data, the model captures posture-relevant features and representations, effectively applying them to radar-related tasks. The key advantage lies in utilizing labeled camera data to obviate the need for direct training on unlabeled radar data, thus mitigating the necessity for annotated radar data.

This cross-modal supervision approach maximizes shared information between radar and camera modalities, allowing knowledge acquired in the camera modality to positively influence radar modality tasks. As a result, it enhances posture recognition performance in the context of through-wall radar information processing, providing a more effective solution for handling multi-modal data in through-wall radar applications.

### 4.1. Cross-Modal Supervision Method

Estimating human body poses behind walls using radar signals poses a significant challenge due to the absence of annotated data. Annotating human poses directly from radar signals is inherently difficult. To tackle this challenge, this study employed a linear regression approach based on a visual model trained to predict human poses from through-wall radar image data.

**Figure 16:**
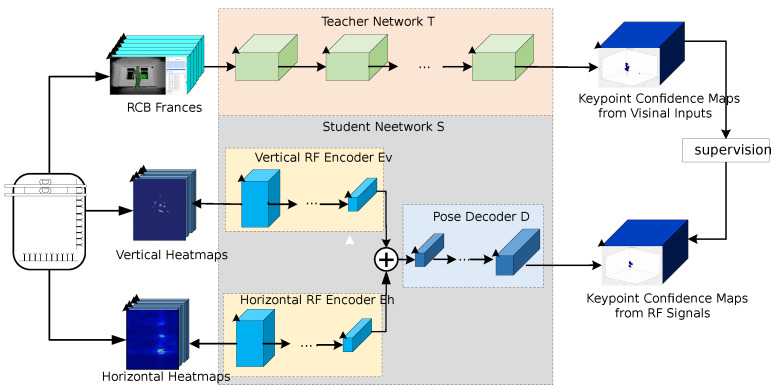
Cross-modal supervision method structure diagram.

This research leveraged a cross-modal teacher–student network [18,19,20] to transfer learned knowledge between the two distinct modalities of the camera dataset and radar dataset. This approach facilitates comprehensive knowledge transfer, including insights into dense keypoint confidence maps [21,22]. The network structure encompasses two pivotal roles: the teacher network and the student network, as depicted in Figure 16. The teacher network is trained on the image modality, while the student network assimilates knowledge transferred from the teacher network regarding radar signals. In the through-wall radar target pose estimation process, dense keypoint confidence maps represent the reliability or confidence of predicted keypoints at each pixel position in the image. Through the cross-modal teacher-student network, more detailed and dense knowledge transfer can be achieved. This methodology is anticipated to enhance the model’s performance across different modalities, enabling a more comprehensive utilization of multi-modal data information in complex tasks such as pose estimation.

This diagram depicts a process divided into two key components. The top process represents the teacher network, which provides cross-modal supervision to the student network in the bottom process, enabling human body pose recognition based on radar signals. The entire network utilizes concurrently acquired images and radar signals as a bridge to transfer visual knowledge of human poses.

In the synchronized images and radar signals for Infrared (I,R), *R* represents the combination of vertical and horizontal heatmaps, while ‘*I*’ corresponds to the respective camera image. The teacher network T(·) taking the camera image ‘*I*’ as input, provides cross-modal supervision to the student network S(·) by predicting the keypoint confidence map T(I). These predicted maps serve as the basis for the student network to learn, enabling it to predict keypoint confidence maps from radar signals. Specifically, a 2D pose estimation network was chosen as the Teacher network in this paper, and the student network focuses on inferring 14 keypoint confidence maps related to the head, neck, shoulders, elbows, wrists, hips, knees, and ankles from radar signals.

The student network S(·) training objective is to minimize the difference between its predictions S(R) and the teacher network’s predictions T(I).

(8) $$\min_{S}\sum_{\left(I,R\right)}L(T(I),S(R))$$

Define the loss as the sum of binary cross-entropy losses for each pixel in the confidence map:

(9) $$L\left(T,S\right)=-\sum_{c}\sum_{i,j}S_{ij}^{c}\log T_{ij}^{c}+\left(1-S_{ij}^{c}\right)\log\left(1-T_{ij}^{c}\right)$$

where $T_{ij}^{c}$ and $S_{ij}^{c}$ are the confidence scores predicted by the teacher network and student network, respectively, for the (i,j) pixel on the confidence map *c*.

This network design has the advantage of transferring the knowledge of the teacher network in the image modality to the network handling radar signals. Through this cross-modal supervision, the model can better understand and leverage information shared between the two modalities. This approach utilizes the existing annotations in camera data during training, thereby circumventing the challenges of direct training on unlabeled radar data and effectively reducing the demand for labeled data.

### 4.2. Human Body Pose Key Point Collection and Input

Given the challenge of acquiring features for posture recognition of targets behind walls, this paper proposed leveraging Kinect in the system to capture individual characteristics in the scene and generate posture information, which can be applied to various recognition tasks. Dynamic models of human body posture naturally represent a series of joint positions over time, expressed in two- or three-dimensional coordinates. Through in-depth analysis of their motion patterns, effective recognition and classification of human posture can be achieved.

Early action-recognition methods primarily formed feature vectors by utilizing joint coordinates at a single time step, followed by temporal analysis of these feature vectors. However, these methods had limited effectiveness, as they did not explicitly consider the spatial relationships between joints, crucial for understanding human behavior. Subsequently, some new methods considering joint connections emerged, showing certain improvements. However, most still relied on manually designed rules for analysis, making them less generalizable [23,24].

This subsection considers the problem of continuous action recognition and introduces a technique based on keypoint estimation and spatiotemporal graph convolution. The core idea of this technique is to establish spatial representations of actions in a continuous sequence by accurately estimating keypoints. Subsequently, through multiple layers of spatiotemporal graph convolution operations, spatial representations are fused with temporal information to generate more advanced spatiotemporal feature maps. Finally, using a standard Softmax classifier, the generated feature maps are precisely classified into corresponding action categories. The uniqueness of this method lies in its ability to effectively capture dynamic information in continuous sequences and accurately classify action categories, having broad prospects for application in the field of continuous action recognition.

#### 4.2.1. Spatiotemporal Graph Convolution

In the graph convolution operation on a single image, under the assumption of a convolution operation with a stride of 1 and appropriate padding, given a convolutional operator of size K × K and an input feature map $f_{in}$ with a channel number of *c*, the output value of a single channel at spatial position *x* can be expressed as

(10) $$f_{out}\left(x\right)=\sum_{h=1}^{K}\sum_{\omega =1}^{K}f_{in}\left(p\left(x,h,\omega \right)\right)\cdot w\left(h,\omega \right)$$

In the graph convolution operation on a single image, assuming a convolutional stride of 1 and appropriate padding, given a convolutional operator of size K × K and an input feature map with a channel number of C, the output value of a single channel at spatial position x can be expressed as follows: where the sampling function *p* is used to enumerate the neighborhood of position *x*, and the weight function ω is utilized to calculate the inner product with the sampled *c*-dimensional channel input feature vector. The adopted formulaic structure is derived from variational convolution. Extending the above equation to the case where the input feature map is represented as a spatial graph $V_{t}$ defines the convolution operation on the graph.

In images, the sampling function refers to the neighboring pixels relative to the central position *x*, representing a region around the central *x* with the size of the convolution kernel. In graph convolution, a similar definition can be applied to the sampling function for the set of neighboring nodes $B\left(v_{ti}\right)=\left\{v_{tj}|d\left(v_{tj},v_{ti}\right)\leq D\right\}$ with respect to the node $v_{ti}$, where $d(v_{ti},v_{tj})$ represents the minimum length (minimum hop count) from $v_{tj}$ to $v_{ti}$. Therefore, the sampling function $p:B(v_{ti})\to V$ can be expressed as

(11) $$P\left(v_{ti},v_{tj}\right)=v_{tj}$$

By setting D=1, denoting the consideration of nearest neighbors in the context of graph convolution, the sampling function p is defined to enumerate the immediate neighboring nodes on the graph, where dtitj represents the minimum hop count from vtj to vti.

In two-dimensional convolution, the spatial order of adjacent pixels is fixed, allowing the weight function to establish indices based on spatial order for element-wise multiplication. In this paper, the spatial order of neighboring nodes is proposed to be determined by a graph labeling process in the neighborhood graph around the root node. This process involves partitioning the neighbor set $B(v_{ti})$ of a certain node $v_{ti}$ into a fixed number of *K* subsets, where each subset shares a common label. Thus, a mapping $l_{ti}:B\left(v_{ti}\right)\to \left\{0,\ldots ,K-1\right\}$ is defined, which maps nodes in the neighborhood to their subset labels. The weight function $w(v_{tj},v_{ti})$ can then be obtained by indexing a (c,K)-dimensional tensor or by:

(12) $$w\left(v_{ti},v_{tj}\right)=w^{\prime }\left(l_{ti}\left(v_{tj}\right)\right)$$

By utilizing the improved sampling function and weight function as described above, $f_{out}\left(v_{ti}\right)$ can be rewritten in the form of graph convolution:

(13) $$f_{out}\left(v_{ti}\right)=\sum_{v_{tj}\in B\left(v_{ti}\right)}\frac{1}{Z_{ti}\left(v_{tj}\right)}f_{in}\left(p\left(v_{ti},v_{tj}\right)\right)\cdot w\left(v_{ti},v_{tj}\right)$$

where the normalization term $Z_{ti}\left(v_{tj}\right)=\left|\left\{v_{tk}|l_{ti}\left(v_{tk}\right)=l_{ti}\left(v_{tj}\right)\right\}\right|$ is equal to the cardinality of the corresponding subset. This term is introduced to balance the contributions of the different subsets to the output. From the above equation, it follows that

(14) $$f_{out}\left(v_{ti}\right)=\sum_{v_{tj}\in B\left(v_{ti}\right)}\frac{1}{Z_{ti}\left(v_{tj}\right)}f_{in}\left(v_{tj}\right)\cdot w\left(l_{ti}\left(v_{tj}\right)\right)$$

It is worth noting that this formula can be made analogous to standard 2D convolution if the image is considered as a regular 2D grid. For example, to resemble 3 ∗ 3 convolution operations, there is a neighborhood of 9 pixels in a grid of 3 ∗ 3 centered on one pixel. The set of neighbors is then divided into 9 subsets, each with one pixel.

#### 4.2.2. Space-Time Modeling

After constructing the graph convolution, it becomes imperative to model the spatio-temporal dynamics inherent in the pose sequence. In the graph construction phase, we introduce the temporal dimension by linking corresponding nodes across successive frames. This straightforward strategy extends the spatial graph convolutional neural network into the spatio-temporal domain by connecting identical nodes across consecutive frames, thereby incorporating temporal relationships. Consequently, the neighborhood concept is expanded to encompass joints interconnected across the temporal domain:

(15) $$B\left(v_{ti}\right)=\left\{v_{qj}|d\left(v_{tj},v_{ti}\right)\leq K,\left|q-t\right|\leq \frac{\Gamma }{2}\right\}$$

The parameter Γ controls the time horizon that is included in the neighborhood graph and can therefore be referred to as the temporal convolution kernel size. In order to realize the convolution operation on the spatio-temporal graph, the sampling function (as in the case of considering only the space) and the weighting function are also needed, in particular the labeled mapping function $l_{ST}$, which makes it straightforward to modify the labeled mapping $l_{ST}$ to the spatio-temporal domain of the root node $v_{ti}$ considering that the temporal axes are ordered, where $l_{ti}$ is the labeled mapping on a single frame, as described before:

(16) $$l_{ST}\left(v_{qj}\right)=l_{ti}\left(v_{tj}\right)+\left(q-t+\frac{\Gamma }{2}\right)\times K$$

#### 4.2.3. Implementation of Spatiotemporal Graph Convolutional Networks

The internal connections of the joints in a single frame are represented by the adjacency matrix *A* and the unit matrix *I* representing the self-connections. In the single-frame case, the spatio-temporal graph convolutional network with the first partitioning strategy can be realized by the following equation:

(17) $$f_{out}=\Lambda^{-\frac{1}{2}}\left(A+I\right)\Lambda^{-\frac{1}{2}}f_{in}W$$

where $\Lambda^{ii}=\sum_{j}\left(A^{ij}+I^{ij}\right)$, the weight vectors of multiple output channels are here superimposed to form a weight matrix *W*.

In practice, considering the spatio-temporal dimension, the input feature map can be represented as a (C,V,T) dimension, and the map convolution operation is realized by performing a standard two-dimensional convolution with a convolution kernel size of 1∗Γ. The tensor obtained is then multiplied by the normalized neighborhood matrix in the second dimension and multiplying the resulting tensor with the normalized adjacency matrix $\Lambda^{-1/2}\left(A+I\right)\Lambda^{-1/2}$ in the second dimension.

For partitioning strategies containing multiple subsets, such as the above distance partitioning and spatial structure partitioning strategies, the above equation is slightly modified, at which time the adjacency matrix is decomposed into multiple matrices, where $A+I=\sum_{j}A_{j}$, for example, in the distance partitioning strategy, satisfies both $A_{0}=I$ and $A_{1}=A$. Then, the above equation is transformed into

(18) $$f_{out}=\sum_{j}\Lambda_{j}^{-\frac{1}{2}}A_{j}\Lambda_{j}^{-\frac{1}{2}}f_{in}W_{j}$$

where $\Lambda^{ii}=\sum_{k}(A_{j}^{ik})+\alpha$, here making α=0.001, avoids blank lines in $A_{j}$. To comprehensively evaluate the efficacy of our proposed method, we conducted a series of corresponding experiments. These experiments encompassed identity recognition tasks performed by multiple subjects across two distinct environments: an open setting where subjects and the radar device shared the same room, and a closed setting with physical barriers separating them. In both environments, subjects were allowed to move freely within the radar’s coverage area, contributing to the dataset utilized for both training and testing purposes.

Upon completing the model training phase, our observations underscored the system’s adeptness in accurately identifying individuals based on continuous sequences of 50 frames of pose heatmaps. It is worth noting that the pose estimation dataset utilized in our study was distinct and separate from the dataset used for identity recognition, ensuring no overlap between the two. For each scenario, a standard CNN model comprising 10 layers was employed for person identification. This deliberate design choice was aimed at bolstering the method’s versatility, thereby ensuring robust and accurate person identification across a spectrum of real-world scenarios.

### 4.3. Keypoint Association and Data Fusion

In the realm of cross-modal supervised human pose recognition, this study diverged from the conventional use of the Euclidean distance metric and instead adopted the Hausdorff distance. This alternative metric was chosen due to its nuanced ability to assess the similarity between captured targets and annotated targets. By leveraging the Hausdorff distance matching algorithm, the system can discern whether the targets depict identical states, thereby enabling precise alignment of human body outlines. This pivotal step aimed to accurately delineate the region of interest, furnishing highly precise matching information for subsequent processing.

While radar reflectograms can provide some degree of through-wall perception for target trajectory localization, the resultant radar imaging often contains significant noise, rendering it unsuitable for trajectory tracking. To address this challenge, this paper introduced a trajectory fitting model that combines keypoints for target trajectory tracking. Additionally, algorithms such as the locally weighted regression smoothing method (Lowess) were employed to smooth the output trajectory from the model.

In terms of keypoint association, our method relies on the keypoint confidence maps generated by the student network for all individuals in the scene, mapping these keypoints onto a skeletal structure. Initially, non-maximum suppression is applied to the keypoint confidence maps to obtain discrete peaks, treated as candidate keypoints. To achieve keypoint association across different individuals, we employed the relaxation method proposed by Cao et al. [25], where the Hausdorff distance is used as the weight between two candidate keypoints. The learned keypoint confidence maps are utilized for frame-by-frame association [26,27].

**Figure 17:**
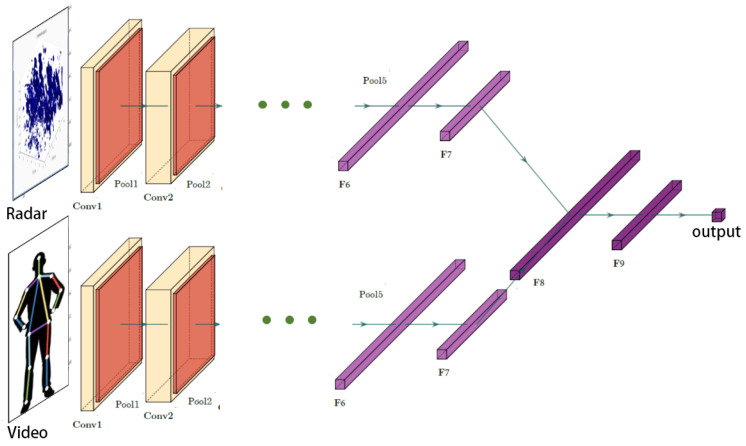
Attitude recognition network model diagram.

For synchronized data acquisition, we conducted collaborative collection of radar and visual data. Cameras were set up in the system, ensuring an average synchronization error not exceeding 10 ms between images and RF data. The radar transmit pattern needed to acquire target information through CNN. Due to the diversity in orientation and position of targets in space, even under the same pose, radar reflection patterns for different targets may exhibit significant differences [28,29]. Therefore, to extract features related to a specific pose of the target and achieve accurate recognition of target pose information, it was necessary to fuse the radar transmit pattern with the human keypoint map, as shown in Figure 17.

The Lowess based algorithm is able to smooth the non-hopping signals, effectively. Therefore, this method was used to filter the recognition results of the network model. For a generic dataset *X*, suppose its *i* input vector is $x_{i}=\left\{x_{i}^{0},x_{i}^{1},\ldots ,x_{i}^{n}\right\}$, where *n* is the data dimension, and the output corresponding to this vector is $y_{i}$, and the number of frames is *m*. For general linear regression, the appropriate weighting parameter, θ, will be chosen so that the loss function is minimized:

(19) $$\min\sum_{i=0}^{m}{\left(y_{i}-\theta^{T}x_{i}\right)}^{2}$$

Unlike focusing on all the data, the locally weighted regression smoothing method focuses more on the distribution of the data near the current prediction point, thus effectively reducing the interference of data far away from the prediction point in the smoothing process. By adding weights $\omega_{i}$ to the loss function, the optimization objective becomes

(20) $$\min\sum_{i=0}^{m}\omega_{i}{\left(y_{i}-\theta^{T}x_{i}\right)}^{2}$$

By adding the weighting information, the loss weights of signals that are farther away from the current position are made lower, thus reducing the impact of the farther away data on the current data. The commonly used weights parameter is

(21) $$\omega_{i}=\exp\left(-\frac{{∥x_{i}-x∥}_{2}^{2}}{2\sigma^{2}}\right)$$

The outputs generated by the deep learning fusion network model, which combines convolutional neural networks with the Lowess smoothing method, could be experimentally compared with traditional filtering algorithms such as the mean filter, median filter, and Kalman filter. This comparative analysis aimed to assess the performance of the deep learning fusion model in trajectory tracking tasks, seeking more precise and dependable trajectory tracking outcomes. Through this comparison with traditional filtering algorithms, we could gain a more comprehensive understanding of the potential advantages offered by the deep learning model in trajectory analysis. This empirical evaluation provided robust support for its effectiveness in real-world applications.

## 5. Experimental Design

### 5.1. Comparative Experiments on Clutter Suppression

#### 5.1.1. Experimental Environment Setup

In this section, experiments were conducted to evaluate the denoising performance of the radar system described in this paper, utilizing SFCW radar signals. The data were processed through individual channels, with a start frequency of 1.9 GHz, an end frequency of 2.9 GHz, a frequency step of 4 MHz, a pulse repetition frequency of 18 Hz, an intermediate frequency (IF) sampling rate of 2 GHz, 1024 points for the inverse fast Fourier transform (IFFT), and a total of 10 × 10 transceiver channels. The system employed a microstrip butterfly oscillator antenna with a back cavity design, which exhibits negligible radiation effects on the human body, as indicated by its parametric specifications.

**Figure 18:**
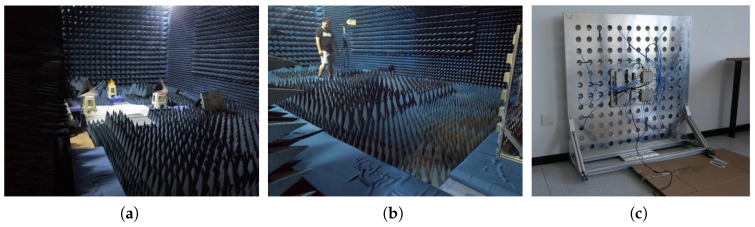
Scene diagram of through-wall radar experiment. (**a**) experimental scenario. (**b**) attitude of personnel. (**b**) radar placement.

The experiments were conducted in a customized microwave darkroom featuring a 12 cm brick wall, ensuring minimal external interference. The radar penetration surface was positioned without any obstruction on PUF foam, further minimizing noise interference. The darkroom environment provided optimal conditions for the experimental observations. The experimental setup is depicted in Figure 18 below.

#### 5.1.2. Comparative Experiments on Clutter Suppression

**Figure 19:**
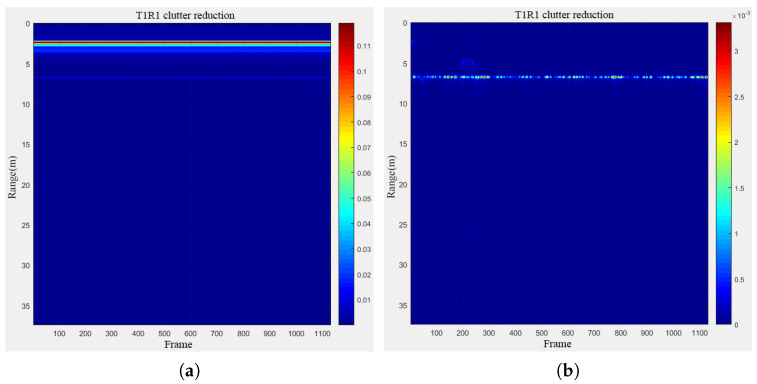
Before and after denoising of stationary human targets: (**a**) original; (**b**) denoising.

Experiments were conducted to image stationary human targets. Initially, a denoising comparison process was performed using a single channel, focusing on a selected set of stationary human targets, as illustrated in Figure 19 below. The experimental results vividly demonstrated that, following the denoising process, these human targets exhibited clear imaging at a distance of 5 m. In contrast, without the denoising process, the targets were barely discernible in the images.

**Figure 20:**
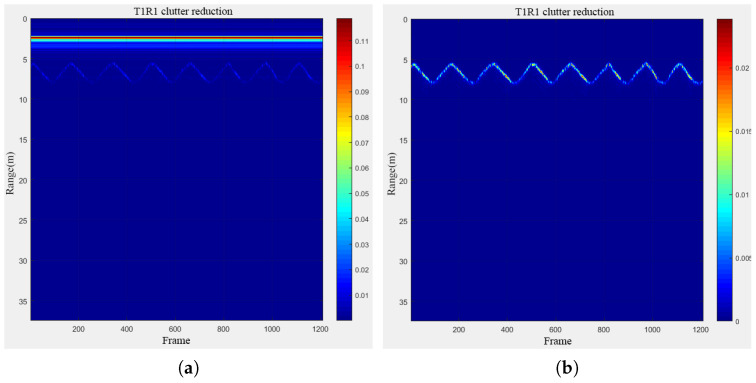
Comparison before and after denoising of sports human targets: (**a**) original; (**b**) denoising.

A group of human targets in longitudinal motion were subsequently chosen for imaging and denoising, as depicted in Figure 20 below. As evident from the figure, at a distance of 5 m, the denoised target imaging demonstrated superior clarity and legibility compared to the non-denoised scenario.

**Figure 21:**
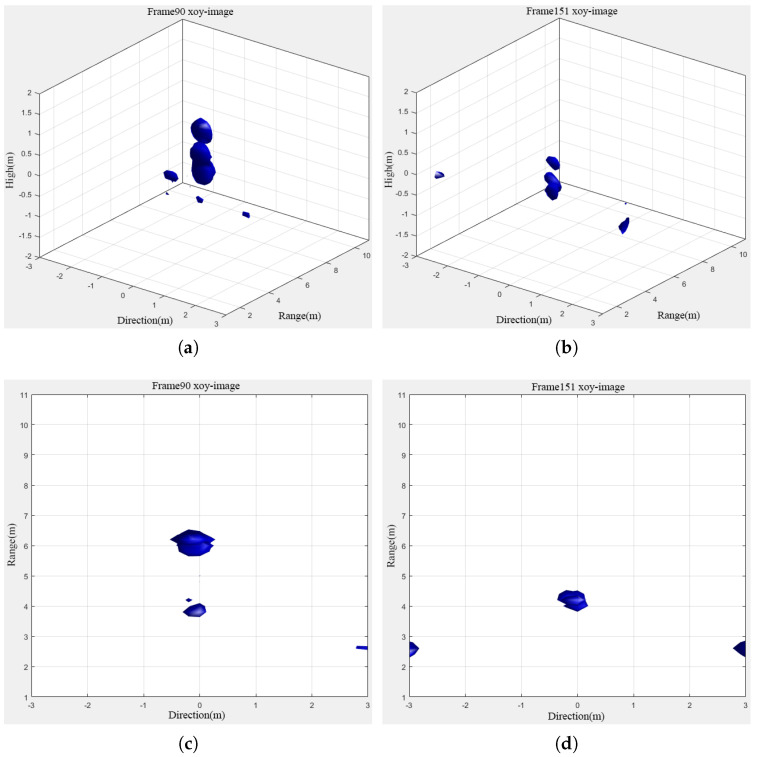
Three-dimensional result map of the moving human target. (**a**) Target movement to (0,5) in three dimensions. (**b**) Target movement to (0,4) in three dimensions. (**c**) Target movement to (0,5) in two-dimensions. (**d**) Target movement to (0,4) in two-dimensions.

The experimental results after denoising were processed using the BP algorithm for 2D to 3D reconstruction. Through this process, this paper successfully realized three-dimensional motion visualization of the target, so that the human naked eye could clearly distinguish these motion trajectories. The results of the human 3D motion experiment are shown in Figure 21 below.

Based on the 3D result map, it is evident that the human target’s contour is remarkably clear in the image. Notably, wall clutter and ghost clutter were effectively filtered out, allowing for a distinct visualization of the target in the top view. Furthermore, the target’s movement from a distance of 5 m to 4 m was accurately captured and depicted. This observation aligns with the 2D distance information provided in Figure 20, underscoring the reliability and precision of the proposed method, particularly in multi-dimensional data processing and target tracking.

### 5.2. Cross-Modal Target Attitude Recognition Experiment

#### 5.2.1. Data Creation

This section outlines the methodology for constructing the essential data samples required for the four-dimensional information denoising technique in through-the-wall radar applications. This encompasses the design of diverse experimental scenarios spanning various through-the-wall radar application contexts and environmental settings, both indoor and outdoor, and involving a wide array of materials and obstacle types. This diversity ensured a thorough evaluation of the algorithm’s efficacy across different scenarios.

Subsequently, the through-wall radar devices were strategically positioned at varied locations and angles to facilitate comprehensive data collection. Through extensive radar imaging, a substantial volume of radar data containing signals from target objects behind obstacles was acquired.

To augment the realism of the target data, a camera was deployed to capture images of the targets. The camera was carefully aligned with the radar equipment to maintain consistent views and positions. Manual labeling of target objects in the camera images, including their position, pose, and key point information, was conducted to provide authentic ground truth data for validating and assessing algorithm accuracy.

During the dataset construction phase, diverse target objects, actions, and poses were selected for experimentation, and data collection was conducted under various environmental conditions to ensure data diversity and richness. Additionally, to simulate real-world application challenges, the dataset was deliberately infused with a range of noises and disturbances.

Following data collection, meticulous organization and labeling were carried out to ensure data consistency and usability. Each data sample was annotated with relevant labels and metadata, facilitating subsequent research and analysis. Through this rigorous data collection and organization process, a high-quality through-the-wall radar 4-D information denoising dataset was successfully curated and made available as open-source. This dataset comprises a wealth of scenarios and target samples, serving as a valuable resource for researchers to investigate and evaluate through-the-wall radar information denoising techniques.

#### 5.2.2. Experimental Environment

**Figure 22:**
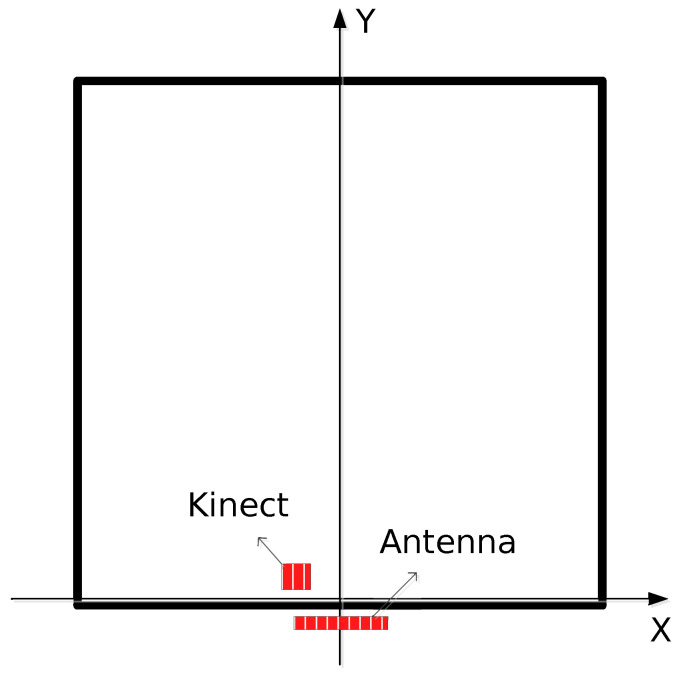
Radar acquisition schematic diagram.

The real test experiment used a 10-transmitter and 10-receiver through-the-wall radar front-end unit, arranged in an ordinary empty room in 6 m ∗ 6 m. The through-wall radar was placed on the outside of the room close to the wall, the specific arrangement is shown in Figure 22 below. There was no obstructions between the target and the wall. It was sufficient for the target to make the prescribed required movements at a distance of 2 m from the wall.

**Figure 23:**
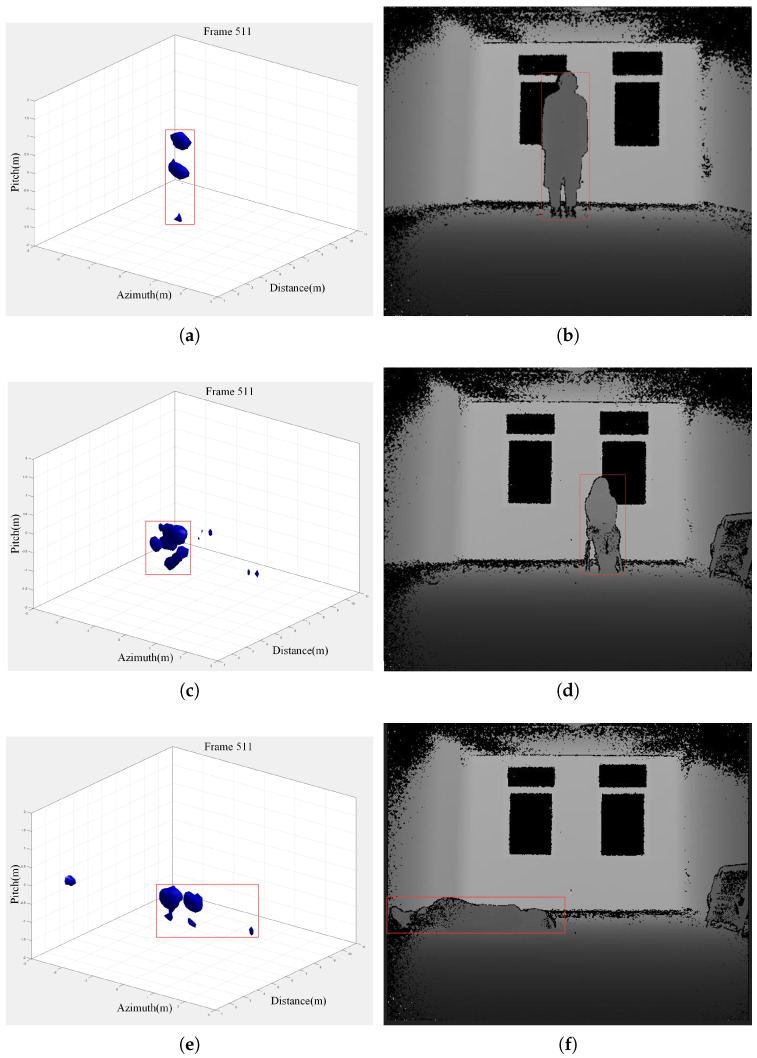
Multiple pose imaging comparison diagram. (**a**,**b**) Standing pose imaging comparison diagram. (**c**,**d**) Sitting pose imaging comparison diagram. (**e**,**f**) Lying pose imaging comparison diagram.

We collected a large amount of static data for three behaviors: standing, sitting, and lying. The data were precisely annotated to the second by personnel for subsequent pose detection. Three sets of typical radar 3D images were selected for comparison. Depth images captured using a Kinect device indoors were used for visual comparison. The images for identification are shown in Figure 23.

Each set of data was as follows: 1000 frames of data were collected for each of the standing pose (a), sitting pose (b), lying pose (c), and background, and a total of 4386 frames were collected. At the same time, supervised data modeling was performed, and a vector of size 4386 ∗ 1 was constructed as the supervised labels, with the standing pose labeled 0, the sitting pose 1, the lying pose 2, and the blank background 3. A total of 300 G of data points were subsequently collected according to this type of scenarios, respectively, for the single-person target, the two-person target, the single-person-plus-interference target, and the action target, and the data have been made available open-source.

#### 5.2.3. Experimental Results

The experiments first trained and detected using traditional machine learning models, and then CNN networks were used for the recognition of static poses, with 5-fold cross-validation, and the evaluation criterion was Classification accuracy. Classification accuracy is defined as

(22) Classificationaccuracy=numberofcorrectlyclassifiedsamples/numberofallsamples

Meanwhile, a confusion matrix was used in the experiments to evaluate the accuracy of the model in classifying different label types. The traditional machine learning methods of SVM, decision tree, random forest, and Adaboost were used to complete the classification experiments for static pose recognition in the room. Finally, a CNN network was used to construct a static pose recognition network for pose recognition. The specific experimental results are shown below:

SVM is a classical supervised machine learning modeling method, which treats the N-dimensional feature vector to be classified as a point in an N-dimensional space, and the goal is to find an optimal N-1 dimensional hyperplane such that the hyperplane can linearly segment N-dimensional feature vectors with different labels.The optimization objective function of SVM is

(23) $$\left[\frac{1}{n}\sum_{i=1}^{n}\max(0,1-y_{i}\left(\vec{\omega }\cdot {\vec{x}}_{i}-b\right))\right]+\lambda {∥\vec{\omega }∥}^{2}$$

where $y_{i}$ and $x_{i}$ are the supervised labels and feature vectors, respectively, ω and *b* are the weights and biases of the SVM classifier, and λ are the hyperparameters. In this experiment, an SVM classifier based on the RBF kernel function was used. The inner product of vectors in this vector space is defined as

(24) $$k\left({\vec{x}}_{i},{\vec{x}}_{j}\right)=\exp\left(-\gamma {∥{\vec{x}}_{i}-{\vec{x}}_{j}∥}^{2}\right)$$

**Figure 24:**
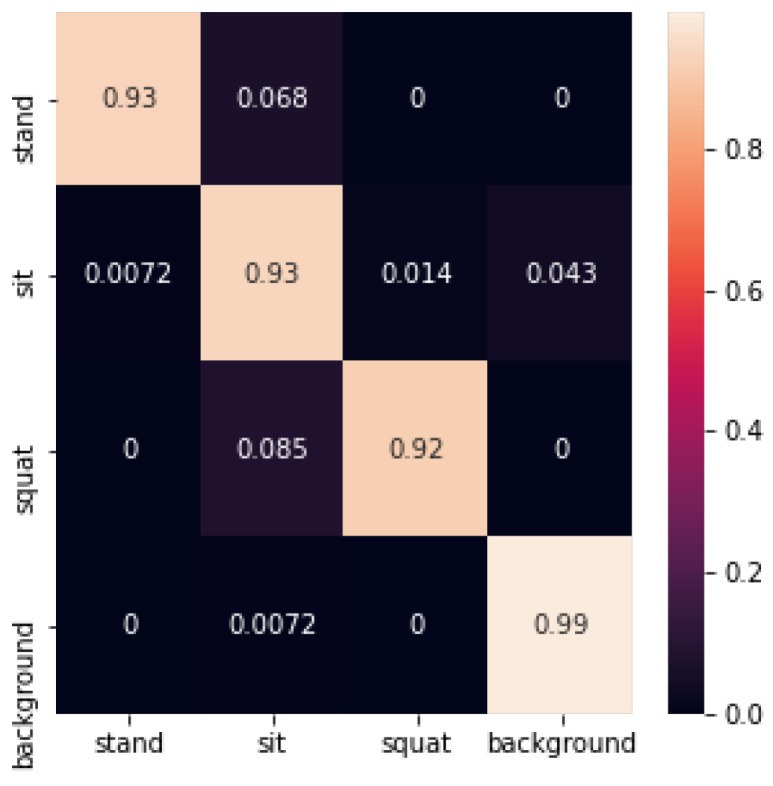
Confusion matrix for SVM recognition.

**Figure 25:**
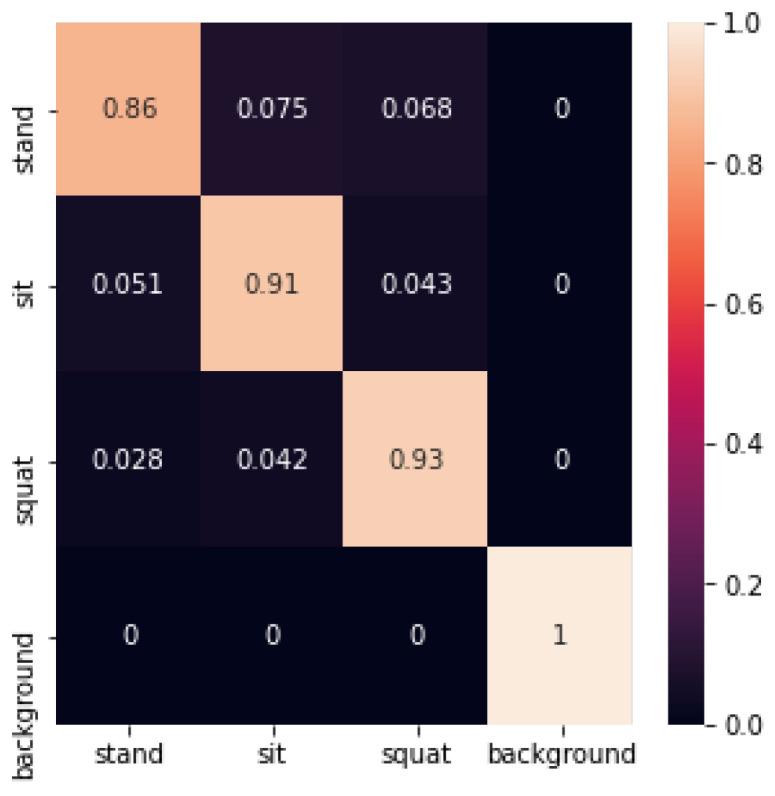
Confusion matrix for decision tree recognition.

The SVM identification confusion matrix is shown below in Figure 24:

The recognition accuracy using SVM is shown in Table 1 below:

Decision trees are a common method in data mining and machine learning. For a given training set, a decision tree is constructed in such a way that the training set is partitioned into subsets based on specific metrics. The commonly used metric is Gini impurity. Gini impurity is defined as follows:

(25) $$I_{G}\left(p\right)=\sum_{i=1}^{J}\left(p_{i}\sum_{k\neq i}p_{k}\right)=\sum_{i=1}^{J}p_{i}\left(1-p_{i}\right)=\sum_{i=1}^{J}\left(p_{i}-p_{i}^{2}\right)=\sum_{i=1}^{J}p_{i}-\sum_{i=1}^{J}p_{i}^{2}=1-\sum_{i=1}^{J}p_{i}^{2}$$

where *J* represents the number of classes, i∈{1,2,…,J}, and $p_{i}$ denote the proportion of samples in the set that belong to the *i*-th class. The decision tree identification confusion matrix is shown below in Figure 25:

The decision tree recognition accuracy is shown in Table 2 below:

In this experiment, the average accuracy of machine learning using decision trees to build machine learning reached 0.92. The accuracy of the standing posture was lower, at 0.86. The recognition accuracy of the sitting posture, squatting posture, and empty background reached 0.91, 0.93, and 1, respectively.

Random forest is a machine learning method based on integrated learning. The basic idea is to construct a large number of decision trees during training and use the average of the output decisions of all the decision trees during decision-making. The biggest advantage of random forest over ordinary decision trees is that it can improve the overfitting tendency of decision trees.

**Figure 26:**
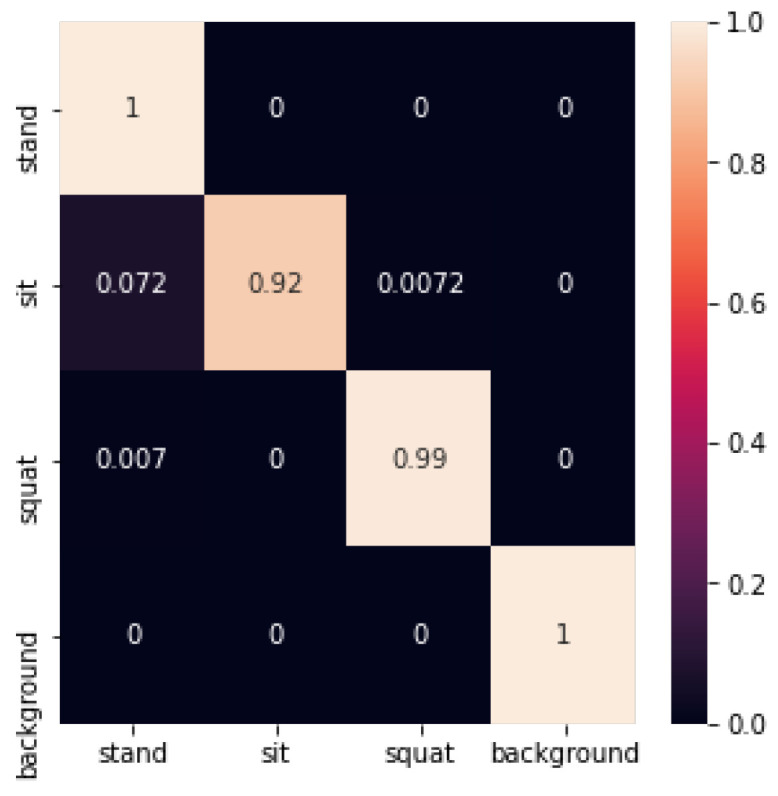
Confusion matrix for random forest recognition.

The random forest identification confusion matrix is shown in Figure 26 below:

The random forest recognition accuracy is shown in Table 3 below:

In this experiment, the random forest method not only improved the performance compared to decision trees, but also demonstrated performance advantages compared to all other methods. Its classification accuracy reached 0.98 on average, with standing, sitting, squatting, and background reaching 1, 0.92, 0.99, and 1, respectively.

Adaboost is another integrated learning method, whose basic idea is similar to that of random forest—constructing a large number of weak classifiers and combining them into a stronger classifier. And it can be shown that the upper bound on the training loss of Adaboost can be decreased exponentially.

**Figure 27:**
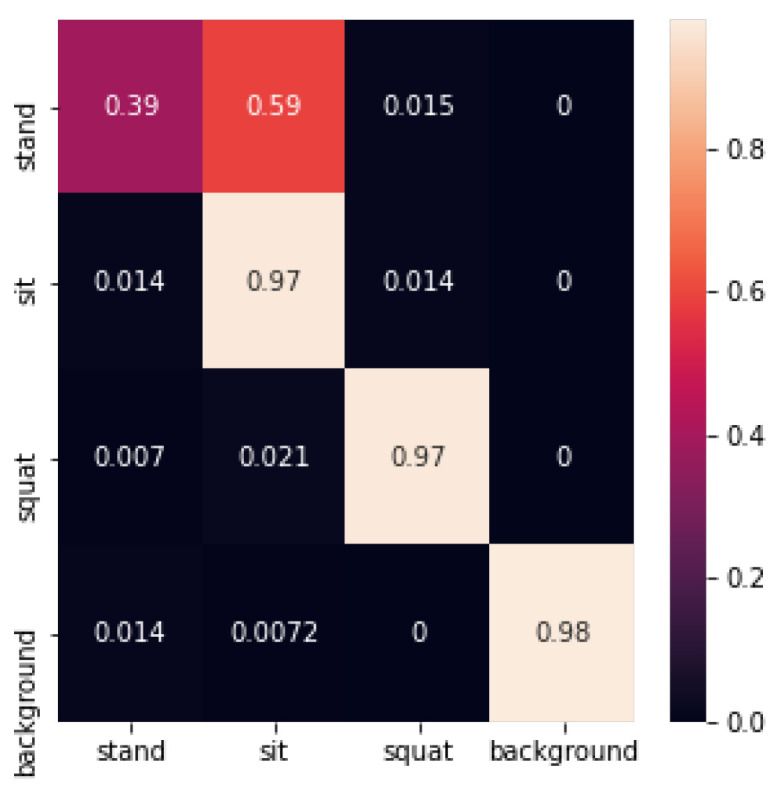
Confusion matrix for Adaboost recognition.

The Adaboost recognition confusion matrix is shown in Figure 27 below:

The Adaboost recognition accuracy is shown in Table 4 below:

In this experiment, the Adaboost method had a low classification accuracy of 0.39 for standing postures. From the confusion matrix, Adaboost had difficulties in distinguishing between standing and sitting postures. From the results of cross-validation, the classification performance of Adaboost was not stable, which suggests that the Adaboost method may have undergone overfitting on the dataset of this test.

NN is one of the most basic and also popular neural network models in the current deep learning field. In this experiment, the convolutional neural network we constructed included a convolutional layer, a pooling layer, and a fully connected layer. The convolutional layer was mainly used for feature extraction, while the fully connected layer was used to perform classification tasks. CNN training was performed using a 8:1:1 training:validation:test set size division. There were 50 epochs of training, a SGD optimizer with a learning rate of 1 × 10^−6^ and cross-entropy loss function.

**Figure 28:**
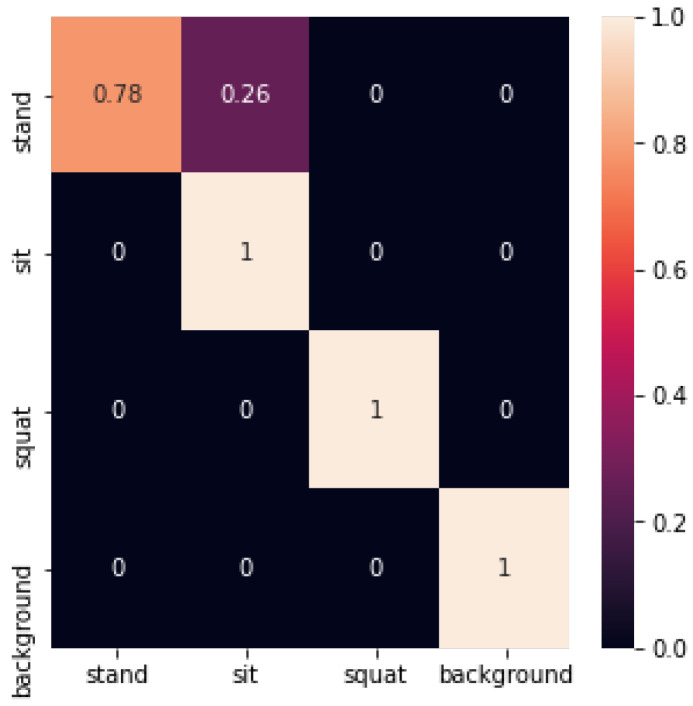
Confusion matrix for recognition by CNN network.

The confusion matrix recognized by the CNN network is shown in Figure 28 below:

The convolutional neural network achieved an accuracy of 0.93 on the test set. It is worth noting that the convolutional neural network achieved better results for the recognition of the sitting posture, squatting posture, and empty background. However, it often recognized the standing posture as the sitting posture.

**Figure 29:**
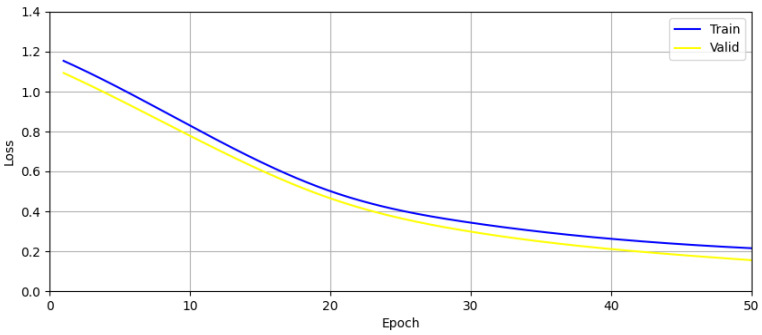
Graph showing the training and validation set losses during CNN network training.

The variation in training set loss and validation set loss of CNN network is shown in Figure 29 below. This figure shows that the training of the convolutional neural network did not suffer from overfitting.

It can be seen that the convolutional neural network approach likewise showed superior potential. Since convolutional neural networks do not require hand-designed feature extraction, this will make it advantageous for classification on larger datasets with unknown features afterwards. In this section, a machine learning approach was proposed to accomplish target attitude detection in MIMO-SFCW radar, and by comparing several traditional machine learning methods, it was demonstrated that it is completely feasible to accomplish multi-dimensional target attitude detection with machine learning.

## 6. Discussion

In this paper, we explored human pose recognition technology under cross-modal supervision and its application to a wall-penetrating radar system. We introduced the theoretical framework for target pose recognition and matching behind a wall using through-wall radar, exploring algorithms for human pose estimation and matching. By amalgamating existing techniques and methodologies and leveraging the unique characteristics of MIMO array wall-penetrating radar, we proposed a cross-modal supervision-based human pose recognition technique tailored for wall-penetrating radar systems. This technique employs deep neural networks to extract high-dimensional features from imaging results, enabling key point detection, attitude recognition, and positional trajectory tracking of the target. The radar’s 3D reflection map and human body key point map are harnessed for target identification and matching. Through an analysis of structural features in the RF signals, we conducted pose segment training to facilitate the recognition of diverse individual body structures. The experimental findings demonstrated that the classifier achieved a recognition rate exceeding 83% for individuals in both visible and through-wall scenarios, with the available data being open-sourced for further research and development.

## 7. Conclusions

The primary focus of this paper revolved around recognizing human body postures concealed behind a wall, leveraging the capabilities of an MIMO array radar system. This study integrates MIMO radar array imaging techniques with deep learning methodologies to address the challenge posed by traditional radar systems, which often struggle to identify human targets due to reflection interference when penetrating walls. By gathering real-world data in wall-penetrating scenarios and utilizing radar 3D reflectograms obtained in previous chapters, a deep learning network was deployed to extract pertinent signals and feature information from noisy 3D reflectograms. These extracted features were then matched with key points on the human body target, enabling the detection of key points, attitude recognition, and trajectory tracking, despite the obstacles presented by wall penetration.

## Figures and Tables

**Table 1:** SVM recognition accuracy.

Test Set	1	2	3	4	5	Average
Accuracy	0.95	0.98	0.92	0.98	0.90	0.94

**Table 2:** Decision tree recognition accuracy.

Test Set	1	2	3	4	5	Average
Accuracy	0.93	0.91	0.92	0.95	0.91	0.92

**Table 3:** Random forest recognition accuracy.

Test Set	1	2	3	4	5	Average
Accuracy	0.99	0.98	0.99	1.00	0.92	0.98

**Table 4:** Adaboost recognition accuracy.

Test Set	1	2	3	4	5	Average
Accuracy	0.98	0.76	0.94	0.74	0.72	0.83
